# Medical Aid in Dying in New York: Clinical, Ethical, and Cardiovascular Considerations Under Assembly Bill A136

**DOI:** 10.7759/cureus.108809

**Published:** 2026-05-13

**Authors:** Philip Branigan, Vo Nhu Y Duong

**Affiliations:** 1 Cardiology, State University of New York (SUNY) Downstate College of Medicine, Brooklyn, USA; 2 Cardiovascular Research, Methodist Hospital, Merrillville, USA

**Keywords:** bioethics, end-of-life care, health policy, heart failure, medical aid in dying, physician-assisted death

## Abstract

New York Assembly Bill A136, scheduled to take effect in August 2026, establishes a statutory framework for medical aid in dying (MAID) for eligible patients with terminal illness. This review synthesizes evidence from established U.S. MAID jurisdictions, including Oregon, Washington, California, Colorado, Vermont, and Montana, with emphasis on clinical implementation, ethical considerations, and cardiology-specific challenges. Although MAID legislation has primarily been discussed in oncology and palliative medicine, its implications for patients with advanced cardiovascular disease have received comparatively less attention in the literature. Heart failure is often characterized by fluctuating disease trajectories, recurrent hospitalizations, prognostic uncertainty, and complex decisions involving advanced therapies and implantable cardiac devices, all of which may complicate assessment of eligibility under the law.

This narrative review examines the major provisions of A136. It also discusses their relevance to cardiovascular practice, with particular attention to prognostication, informed consent, palliative care integration, clinician responsibilities, and ethical distinctions between MAID and withdrawal of life-sustaining therapies. The review also considers how social determinants of health and disparities in cardiovascular care may influence patient experiences, symptom burden, and end-of-life decision-making. As MAID becomes integrated into clinical practice in New York, cardiologists and multidisciplinary care teams will need to balance legal requirements with careful communication, individualized patient care, and established principles of cardiovascular palliative medicine.

## Introduction and background

**Figure 1:**
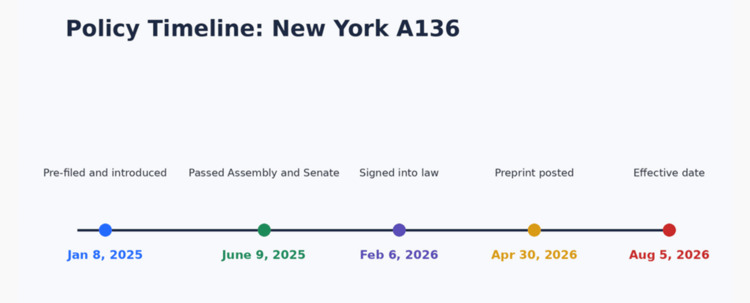
Policy timeline: New York Assembly Bill A136 Timeline of key legislative milestones for Assembly Bill A136 during the 2025-2026 session, including introduction, passage, enactment, and scheduled implementation. The figure highlights the sequence of events relative to the timing of this review. Created using Microsoft PowerPoint (Microsoft Corporation, Redmond, WA)

Medical aid in dying (MAID) has evolved over several decades in the United States, with multiple states adopting statutory frameworks that permit physician-assisted death under defined conditions. New York Assembly Bill A136, introduced during the 2025-2026 legislative session, was pre-filed on January 8, 2025, and passed both the State Assembly and Senate on June 9, 2025. Following additional negotiations regarding procedural safeguards, the bill was signed into law by Governor Kathy Hochul on February 6, 2026, and is scheduled to take effect on August 5, 2026 (Figure 1) [1]. The legislation defines MAID as a physician prescribing medication that a qualified individual may self-administer to end life, explicitly excluding administration by any other person and prohibiting lethal injection or infusion.

Eligibility under A136 requires that the patient be an adult with decision-making capacity, diagnosed with a medically confirmed terminal illness expected to result in death within six months, and making a voluntary and informed decision free from coercion [1]. While these criteria resemble those in other jurisdictions and may raise similar concerns [2-5], the New York statute provides more granular procedural detail, particularly regarding documentation, witness requirements, and institutional responsibilities. This review examines the anticipated implementation of A136 with attention to clinical practice and cardiology-specific considerations [6-8].

This article was previously posted to the SSRN preprint server on April 30, 2026.

## Review

Methods

Study Design and Time Frame

This study was conducted as a narrative review with a structured approach to identifying and synthesizing relevant literature. The review was performed between January 2026 and April 2026, after legislative passage but prior to the law’s effective date, and was intended to contextualize New York Assembly Bill A136 using data from jurisdictions with established MAID frameworks.

Data Sources and Search Strategy

A focused literature search was carried out using PubMed/MEDLINE, Embase, Scopus, and Google Scholar. Additional sources included state legislative materials and policy statements from professional organizations.

The search was limited to publications from January 2000 through April 2026 to reflect modern MAID practice. Keywords were combined using Boolean operators and included:

“medical aid in dying” OR “physician assisted death” OR “assisted suicide” AND “implementation” OR “policy” OR “legislation” AND “Oregon” OR “Washington” OR “California” OR “Colorado” OR “Vermont” OR “Montana” OR “Canada” AND “ethics” OR “clinical practice” OR “physician attitudes” AND “heart failure” OR “cardiology” OR “palliative care” OR “end of life care”.

Reference lists of selected articles were reviewed to identify additional relevant sources.

Eligibility Criteria

Inclusion criteria: Sources were included if they were peer-reviewed articles, clinical guidelines, or official legal or policy documents; examined MAID or physician-assisted death in established jurisdictions such as Oregon, Washington, California, Colorado, Vermont, Montana, or Canada; addressed clinical processes, ethical considerations, physician experience, or health system implementation; included relevance to cardiovascular disease or palliative care when available; were published in English.

Exclusion criteria: Sources were excluded if they focused on euthanasia practices outside comparable legal frameworks; were primarily opinion-based without supporting clinical or empirical data; addressed proposed legislation without implementation experience; were duplicates or not accessible in full text.

Study Selection

The search strategy was designed to identify a focused and relevant body of literature rather than to be exhaustive. After screening for relevance and removing duplicates, 18 sources were selected for inclusion, corresponding to the references cited in this review. These included observational studies, survey-based research, clinical guidelines, and legal or policy analyses.

Data Extraction and Synthesis

Information from included sources was reviewed with attention to jurisdictional structure, clinical workflows, physician perspectives, ethical considerations, and issues specific to cardiovascular disease such as prognostic uncertainty in heart failure.

Given the variability in study design and the inclusion of legal and policy materials, findings were synthesized qualitatively rather than through formal quantitative analysis.

Rationale for Approach

The approach was chosen to integrate legal, clinical, and ethical perspectives in a way that would be useful for clinicians preparing for the implementation of A136. Emphasis was placed on established MAID programs to reflect practical experience. Cardiology was included as a focus because disease trajectory and prognostication differ from other terminal conditions and may influence how eligibility criteria are applied in practice.

Legal and procedural framework

Assembly Bill A136 establishes a multi-step request and verification process codified in Sections 2899-e through 2899-j. A patient must make both an oral request and a written request, the latter signed, dated, and witnessed by two adults who meet strict eligibility criteria designed to prevent conflicts of interest. The statute prohibits relatives, potential heirs, and facility employees from serving as witnesses, reinforcing safeguards against coercion [1].

The attending physician must determine that the patient meets all eligibility criteria, including terminal illness, decision-making capacity, voluntariness, and informed decision-making. A consulting physician must independently confirm the diagnosis, prognosis, and capacity assessment before a prescription may be written. If either physician suspects impaired judgment, referral to a qualified mental health professional is mandatory, and a finding of impaired capacity disqualifies the patient [1].

The law also requires extensive documentation in the medical record, including all requests, clinical determinations, and confirmation that statutory steps have been followed. Patients retain the right to rescind their request at any time and in any manner, and the attending physician must explicitly offer an opportunity to rescind before prescribing medication [1].

Clinician participation is voluntary. Section 2899-m affirms that no physician or health care provider is required to participate, and facilities may prohibit MAID within their institutions if based on formally adopted policies grounded in moral or religious principles, provided patients are informed, and transfers are arranged when appropriate. These provisions align with national patterns emphasizing clinician autonomy [4].

Clinical communication and patient-centered care

A136 places significant emphasis on informed decision-making, requiring explicit discussion of diagnosis, prognosis, risks of the medication, expected outcomes, and feasible alternatives, including palliative and hospice care. The statute goes further by mandating that physicians provide state-developed, culturally appropriate educational materials on end-of-life care when available [1].

Requests for MAID under this framework must be understood in context. As observed in other jurisdictions, such requests often reflect concerns about suffering, autonomy, or loss of control rather than a singular desire for death [9]. The statutory requirement to review alternatives creates a structured opportunity to address unmet needs through symptom management, psychosocial support, or hospice referral.

Data from Colorado suggest that while physicians are generally willing to engage in these discussions, many feel underprepared [5]. Given the detailed counseling obligations under A136, gaps in clinician training may become more pronounced in New York without targeted educational efforts.

Institutional readiness and implementation

The operational demands of A136 are substantial. Health systems must develop policies that address each statutory requirement, including request tracking, documentation workflows, consultation pathways, and pharmacy coordination for medication dispensing. The law also introduces requirements for safe disposal of unused medications and mandates annual reporting and review by the state health commissioner.

Experience from California and Canada indicates that centralized coordination models can facilitate compliance and reduce burden on individual clinicians [7,10]. Under A136, similar structures may be necessary to ensure that attending and consulting roles, mental health evaluations, and documentation standards are consistently applied.

Education will be critical. Physicians must be trained not only in end-of-life communication but also in the specific legal thresholds embedded in the statute, including the definition of “informed decision,” witness eligibility, and documentation requirements. Current gaps in training identified in residency and continuing education literature highlight the need for proactive curriculum development [11,12].

Ethical considerations

Assembly Bill A136 attempts to draw clear ethical and legal boundaries. It explicitly states that actions taken in accordance with the statute do not constitute suicide, assisted suicide, or homicide under New York law [1]. This legal framing is intended to distinguish MAID from other end-of-life practices and to provide protection for participating clinicians.

At the same time, the ethical tension between autonomy and nonmaleficence remains. The statute emphasizes voluntariness, informed consent, and the availability of alternatives, reflecting core bioethical principles. The requirement that patients self-administer medication reinforces the distinction between MAID and euthanasia.

**Figure 2:**
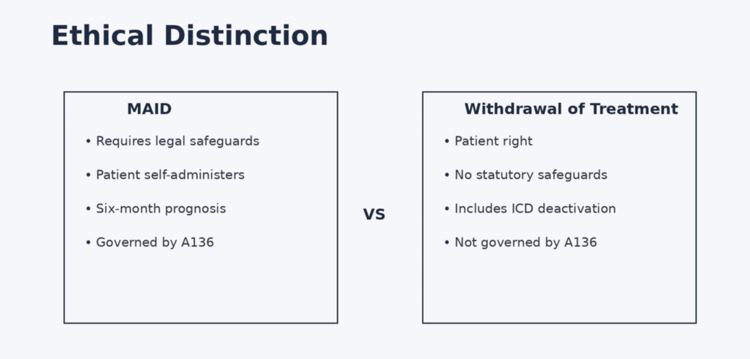
Distinction between medical aid in dying and withdrawal of life-sustaining treatment The figure highlights the distinction between a regulated process under Assembly Bill A136, which requires defined safeguards and patient self-administration, and withdrawal of treatment, which is a patient right based on informed refusal and is not governed by the statute. Created using Microsoft PowerPoint (Microsoft Corporation, Redmond, WA) ICD: implantable cardioverter-defibrillator

Equally important is the distinction between MAID and withdrawal of life-sustaining treatment. The latter is recognized as a patient right and does not require the procedural safeguards outlined in A136 (Figure 2). Maintaining clarity between these practices is essential to avoid confusion among clinicians and patients [2].

Cardiovascular disease and MAID

**Figure 3:**
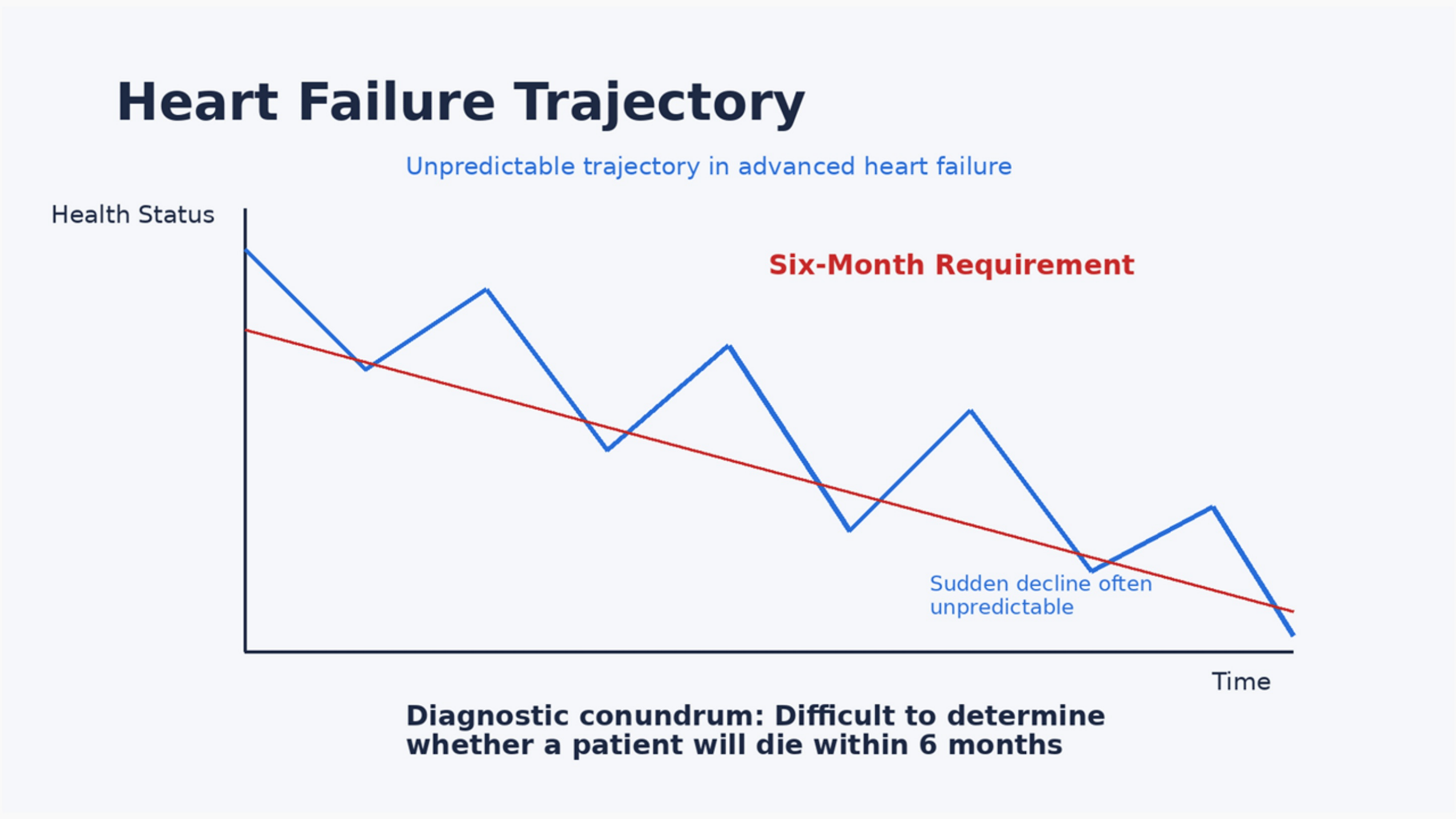
Heart failure trajectory and six-month prognosis requirement Illustration of the clinical course of advanced heart failure in relation to the six-month prognosis requirement in Assembly Bill A136. The figure highlights the variable trajectory and the uncertainty around predicting short-term mortality. Created using Microsoft PowerPoint (Microsoft Corporation, Redmond, WA)

A136’s eligibility requirement of a six-month prognosis presents particular challenges in cardiovascular disease. Heart failure trajectories are often unpredictable, with periods of stability interrupted by acute decompensation (Figure 3). Determining whether a patient meets the statutory definition of terminal illness may therefore be more complex than in oncology populations [13-16].

The law requires that the prognosis be based on reasonable medical judgment and confirmed by a consulting physician. Prognostic tools, such as the Seattle Heart Failure Model, and data from trials including PARADIGM-HF and EMPEROR-Reduced can inform this judgment but cannot eliminate uncertainty [5,8,17]. Clinicians must communicate this uncertainty transparently while still meeting statutory requirements.

Prognostic uncertainty in heart failure may also intersect with broader disparities in cardiovascular care. Patients from underserved communities often experience higher symptom burden, repeated hospitalizations, delayed access to specialty care, and limited access to palliative services. In a recent New York City cohort of patients living with HIV and heart failure, Black and Hispanic patients were more likely to live in disadvantaged neighborhoods and report social adversity, while differences in procedural management and mortality were also observed [18]. These factors may influence how patients experience advanced illness and approach end-of-life decisions, including discussions surrounding MAID.

Triggers for MAID discussions in heart failure

Although A136 does not specify clinical triggers for MAID discussions, its structure implies that such conversations will often arise in the context of advanced disease and significant symptom burden. Common triggers include refractory symptoms, repeated hospitalizations, and patient-initiated requests for hastened death [3].

The statute requires that alternatives such as palliative and hospice care be discussed in all cases, reinforcing the role of supportive care as a parallel pathway rather than a prerequisite or substitute. Evidence supports early integration of palliative care in heart failure to improve quality of life and psychological outcomes [6,13-15].

Cardiac device management and ethical distinctions

The detailed procedural framework of A136 may help clarify distinctions that are often blurred in clinical practice. Deactivation of implantable cardioverter-defibrillators or withdrawal of left ventricular assist device support are considered withdrawal of therapy and are not governed by the statute. In contrast, prescribing medication under A136 requires adherence to the full legal process.

Failure to distinguish these practices can lead to delays in appropriate care or unnecessary ethical conflict. Education that explicitly contrasts device deactivation with MAID will be important as clinicians adapt to the new legal environment [2,4,16].

Provider experience and well-being

A136 includes protections for clinicians who participate or decline participation, shielding them from civil, criminal, and professional liability when acting in good faith [1]. While these protections may reduce legal concerns, the emotional and ethical impact of participation remains significant.

Evidence from other states suggests that clinicians may find participation meaningful but also report moral distress and increased workload [5]. Institutional support structures, including ethics consultation and peer discussion, will be important components of implementation.

Limitations

This review is based on a statute that has not yet been implemented in clinical practice. As a result, many of the operational challenges discussed are inferred from the structure of the law and from experiences in other jurisdictions rather than from direct evidence in New York. Actual implementation may differ depending on institutional policies, resource availability, and evolving state guidance.

The analysis also relies on data from states and countries with established MAID frameworks. While these comparisons are useful, differences in legal language, healthcare infrastructure, and patient populations limit how directly those experiences can be applied to A136. New York’s more detailed procedural requirements may introduce challenges that have not been observed elsewhere.

Cardiology-specific considerations are drawn largely from existing literature on heart failure trajectories, prognostic tools, and palliative care integration. However, there is limited empirical research examining how patients with cardiovascular disease engage with MAID statutes. In particular, the uncertainty of prognostication in heart failure makes it difficult to predict how frequently patients will meet eligibility criteria or pursue this option in practice.

The discussion of clinician preparedness and well-being is similarly constrained by available data. Most evidence comes from survey-based studies in other states, which may not fully capture the perspectives of cardiologists or reflect the impact of New York’s specific legal framework. Local training programs, institutional culture, and access to support services will likely influence clinician experience in ways that cannot yet be measured.

Finally, this review focuses primarily on statutory and clinical dimensions and briefly addresses broader social, cultural, and health equity considerations. Factors such as access to care, socioeconomic status, racial disparities in cardiovascular outcomes, and cultural attitudes toward end-of-life decision-making may significantly influence how A136 is used across different patient populations. In addition, there remains limited real-world data regarding the implementation of MAID in patients with advanced cardiovascular disease, particularly among underserved populations. These issues warrant further study as real-world data become available following implementation of A136 in New York.

## Conclusions

Assembly Bill A136 introduces one of the most detailed statutory frameworks for MAID in the United States, with a clear emphasis on documentation, procedural safeguards, and patient education that reflects the broader national movement toward physician-assisted death laws. Compared with earlier state laws, it provides greater specificity in areas such as witness qualifications, mental health evaluation, institutional participation, and post-prescription responsibilities. For cardiologists, the law shifts MAID from a primarily ethical consideration to a structured and highly regulated clinical process. Implementation may be particularly challenging because advanced heart failure often follows an unpredictable clinical course. Prognostic uncertainty, recurrent hospitalizations, and decisions involving cardiac devices or advanced therapies may complicate assessment of eligibility and end-of-life planning. At the same time, social determinants of health and disparities in cardiovascular care may shape how patients experience suffering and approach these discussions. As MAID becomes integrated into clinical practice in New York, careful communication, interdisciplinary collaboration, and attention to palliative care needs will remain essential in the care of patients with advanced cardiovascular disease. Clinicians will need to become familiar with the legal requirements of Article 28-F while maintaining a focus on patient needs and the multidisciplinary principles of palliative care, respect for clinician autonomy, and clear distinctions between MAID and other end-of-life medical decisions.
